# Too Much Information: Assessing Privacy Risks of Contact Trace Data Disclosure on People With COVID-19 in South Korea

**DOI:** 10.3389/fpubh.2020.00305

**Published:** 2020-06-18

**Authors:** Gyuwon Jung, Hyunsoo Lee, Auk Kim, Uichin Lee

**Affiliations:** ^1^Graduate School of Knowledge Service Engineering, Korea Advanced Institute of Science and Technology, Daejeon, South Korea; ^2^Department of Industrial and Systems Engineering, Korea Advanced Institute of Science and Technology, Daejeon, South Korea

**Keywords:** privacy, contact tracing, COVID-19, data disclosure, personal data, travel log

## Abstract

**Introduction:** With the COVID-19 outbreak, South Korea has been making contact trace data public to help people self-check if they have been in contact with a person infected with the coronavirus. Despite its benefits in suppressing the spread of the virus, publicizing contact trace data raises concerns about individuals' privacy. In view of this tug-of-war between one's privacy and public safety, this work aims to deepen the understanding of privacy risks of contact trace data disclosure practices in South Korea.

**Method:** In this study, publicly available contact trace data of 970 confirmed patients were collected from seven metropolitan cities in South Korea (20th Jan–20th Apr 2020). Then, an ordinal scale of relative privacy risk levels was introduced for evaluation, and the assessment was performed on the personal information included in the contact trace data, such as demographics, significant places, sensitive information, social relationships, and routine behaviors. In addition, variance of privacy risk levels was examined across regions and over time to check for differences in policy implementation.

**Results:** It was found that most of the contact trace data showed the gender and age of the patients. In addition, it disclosed significant places (home/work) ranging across different levels of privacy risks in over 70% of the cases. Inference on sensitive information (hobby, religion) was made possible, and 48.7% of the cases exposed the patient's social relationships. In terms of regional differences, a considerable discrepancy was found in the privacy risk for each category. Despite the recent release of government guidelines on data disclosure, its effects were still limited to a few factors (e.g., workplaces, routine behaviors).

**Discussion:** Privacy risk assessment showed evidence of superfluous information disclosure in the current practice. This study discusses the role of “identifiability” in contact tracing to provide new directions for minimizing disclosure of privacy infringing information. Analysis of real-world data can offer potential stakeholders, such as researchers, service developers, and government officials with practical protocols/guidelines in publicizing information of patients and design implications for future systems (e.g., automatic privacy sensitivity checking) to strike a balance between one's privacy and the public benefits with data disclosure.

## 1. Introduction

With COVID-19 becoming a worldwide pandemic, each country is attempting various ways to stop or slow down the spread of the virus among people, such as social distancing, preventing events that bring many people together, detecting and isolating the confirmed cases, and so on (1).

In this situation, one of the effective measures is to conduct “contact tracing” (1, 2). Contact tracing is defined as “the identification and follow-up of persons who may have come into contact with an infected person,” and involves identifying, listing, and taking follow-up action with the contacts (3). It plays an important role in quick isolation of infected persons to prevent potential contact with others. From a stochastic transmission model of the spread of COVID-19, contact tracing was shown to be effective in controlling a new outbreak in most cases and reducing the effective reproduction number (2).

However, due to limited human resources for tracing, it could be very difficult to trace the contacts who might be potentially infected, particularly when the number of patients is skyrocketing. Therefore, some countries began to proactively open the data of confirmed cases to the public or share it with medical institutions to find close contacts more efficiently. For instance, in Singapore, the government discloses the places related to patients, such as residence, workplaces, and other places they had visited (4). In Taiwan, the authorities utilize the airport immigration database combined with the national medical database to quickly determine whether the patient has visited other countries (5). Other governments also are sharing the personal information of the patients with similar components of data, including age and gender, nationality, geographical breakdown of patients, and so on (6).

South Korea also disclosed the patients' contact trace data to the public to prevent further spread of the coronavirus. Each local government pseudonymizes the patient data, which contains demographics, infection information, and travel logs, and releases it to the public. This information helps the public to self-check whether they were co-located with the confirmed patient. However, there is a potential threat in publicizing the patient's data (7). Efficiently identifying potential contacts may be advantageous in terms of public safety but revealing personal data would infringe upon the patient's privacy. Most of the information disclosed could be personal data and combining a set of data reveals additional information. Privacy risks, along with online abuses or rumor-mongering based on somewhat uncertain information, may cause blame and social stigma (8, 9) and raise the risk of physical safety (10).

While it is important to find and isolate close contacts quickly for preventing the spread of infectious diseases, it is also critical to minimize breach of patients' privacy. Recently, the National Human Rights Commission of Korea claimed that the publicized information is unnecessarily specific and may cause privacy violations (11). In response to this, the Korea Centers for Disease Control & Prevention (hereinafter “KCDC”) announced two guidelines (12, 13) limiting the scope and the period of the data disclosure and recommended the deletion of outdated information (after 14 days from the last contact) on March 14 and April 12, respectively (see Table 1).

**Table 1:** The Korean government Guidelines for the scope and detail of the information to be disclosed.

**Issue date**	**Details of guideline**
Mar. 14	• **Personal information:** Information that identifies a specific person should be excluded
	• **Period:** Information should be from 1 day before the symptoms occur to the date of quarantine
	• **Place and transportation:** Place and transportation should be disclosed where contacts have occurred with the confirmed cases. The detailed address of residence and workplace should not be disclosed. However, the address may be revealed if there is a risk that COVID-19 has been spread to random people in the workplace. Spatial and temporal information (e.g., building, place names, and transportation) should be specified as possible, except for that of identifying certain individuals.
Apr. 12	• **Disclosure period:** the data should be only released for 14 days from the date that the patient had the last contact.

Although a critical question about the cost-benefit tradeoffs between privacy and public safety still remains, existing studies on location and privacy have not fully reported insights from contact tracing and underlying privacy risks. Past studies on location privacy primarily focused on an individual's privacy perceptions and potential risks of leaking current locations to diverse social media (14–16). However, these prior works were more of a real-time location sharing of a single spot, rather than sharing one's full mobility data spanning several days to a week or more, as in contact tracing. Another key difference to note is that privacy risks regarding contact tracing under special occasions, such as COVID-19 are relatively unaddressed in the literature. It is timely to explore this issue as public disclosure of contact tracing data under COVID-19 raises questions about data sovereignty and privacy of a patient. Thus, the present study assessed privacy risks on the contact trace data disclosed in South Korea. Specifically, the study first examined what kind of personal information is contained in the data, and how much exposure or inference is made from that data. It then examined how much difference in privacy risk levels exists according to region and time when disclosing the data. While no study to the researchers' knowledge has assessed privacy risks on public disclosure of contact tracing data related to COVID-19, the present study first analyzes the real-world data in South Korea and provides possible directions for privacy-preserving data disclosure and presents several policy and technical implications that can possibly lower privacy risks.

## 2. Materials and Methods

This section describes the data collection and analysis process used to evaluate privacy issues resulting from data disclosure.

### 2.1. Data Collection

To assess potential privacy concerns through real-world examples, the contact trace data of 970 confirmed patients was collected. The data listing confirmed cases date-wise from January 20 to April 20 were released by seven major metropolitan cities in South Korea.

The contact trace data was collected from various publicly accessible online websites, such as the official website and social media sites of the local government, and its press releases and briefing information. Since the data was released to the public by the government and any specific individual cannot be identified with it, there is no critical ethical concern for data analyses. As shown in Table 2, the released contact trace data included (1) the patient's demographics (i.e., nationality, gender, age, and residence), (2) infection information (i.e., infection route and confirmation date), and (3) travel log in time series (e.g., transport modes and visited places). The data is processed by the contact trace officer before it is released online (i.e., excluding places which the patient visited but no contact was made), hence the government may possess more information than the public can access.

**Table 2:** The contact trace data of a confirmed patient (Patient No. #e06). The contact trace data of other patients from Seoul can be found in Seoul Metropolitan Government (17).

**<#e06th confirmed patient>**		
○ **Patient information:** 50 years old, female, lives in HO3H-dong		
○ **Infection route:** a member of 07ZT-gu 8RYY Church had a direct contact with YH4C-gu confirmed patient (a pastor of 07ZT 8RYY Church) on Mar.19 (Thu)		
○ **The route:**		
*Mar. 31 (Tue)*	[07:05–07:20]	Home->Subway line 2 EJ5B station (on foot), took a subway
	[07:20–10:30]	The route in other district
	[10:30–15:10]	Got off at Subway line 2 EJ5B station/stayed at home after arriving home (on foot)
	[15:10–15:43]	FG87 Outlet (7VGV-ro 2OS9) (on foot)
		*wore mask, no direct contact *disinfection has completed. The place is safe
	[15:43–16:10]	Y2GX Mart (2E4V-ro 8LO8) (on foot)
		*wore mask, no direct contact *disinfection has completed. The place is safe
	[After 16:10]	Stayed home after arriving home (on foot)
*April 1 (Wed)*	[07:05–07:20]	Home ->Subway line 2 EJ5B station (on foot)/took a subway
	[07:20–10:00]	The route in other area
	[10:00–15:00]	Got off at Subway line 2 EJ5B station/stayed at home after arriving home (on foot)
	[15:10–15:36]	FG87 Outlet (7VGV-ro 2OS9) (on foot)
		*wore mask, no direct contact *disinfection has completed. The place is safe
	[15:36–16:06]	Y2GX Mart (2E4V-ro 8LO8) (on foot)
		*wore mask, no direct contact *disinfection has completed. The place is safe
	[16:06–17:00]	Stayed at home after arriving home (on foot)
	[17:00–17:40]	Took a test at 2E4V Health Center, a designated clinic (on foot)
		*with 1 acquaintance(tested negative)
	[After 17:40]	Stayed home after arriving home (on foot)
*April 2 (Thu)*	[07:05–07:20]	Home->Subway line 2 EJ5B station (on foot)/took a subway
	[07:20–10:00]	the route in other district
	[After 10:00]	Got off at Subway line 2 EJ5B station/stayed home after arriving home (on foot)
* Tested positive/transferred to A484 University Hospital		

This study covered seven out of eight metropolitan cities in South Korea, namely, Seoul, Incheon, Sejong, Daejeon, Gwangju, Ulsan, and Busan. The city of Daegu was excluded from the data collection process because it did not disclose patient information since the massive contagion outbreak prevented contact tracing.

As the guidelines set by the KCDC recommend the deletion of the outdated information (after 14 days from the last contact), all the sample cases of disclosed patient data mentioned in this study were anonymized by the researchers. For instance, the address and name of a place (e.g., building name) were converted into four character long random strings (e.g., G3A5-gu, D12Z-dong, BQT3 building). Similarly, the identification number of the patient was also anonymized (e.g., #w4p).

### 2.2. Codebook Generation

In this study, a codebook was introduced to evaluate the level of privacy risks. The codebook has an ordinal scale of privacy risk levels and the scale quantifies relative risks from five major categories: demographics (nationality, gender, age), significant places (residence, workplace), sensitive information (hobby, religion, accommodation), social relationships, and routine behavior. The details of the codebook generation are as follows:

The collected data were manually examined to evaluate the level of privacy risks. The following types of information were identified: demographics, location information (e.g., significant places and behavioral routines), and social relationships. Affinity diagramming on contact trace data was performed to iteratively build a coding scheme (18). As a result, the manual examination generated five categories with eight sub-categories, as described in Table 3. For each data category, an ordinal scale of privacy risk levels was introduced. The scale quantifies the relative privacy risks of the patient's trace data; for example, a high level means that detailed information was released. The following section describes the details of each category and its associated risk levels. This codebook was used to evaluate each patient's contact trace from seven metropolitan cities.

**Table 3:** The ordinal scales of privacy levels across data categories.

**Category**	**Sub-category**	**Privacy levels**	**Description**
Demographics	Nationality/Gender	0	Not disclosed
		1	Disclosed
	Age	0	Not disclosed
		1	Roughly disclosed
		2	Fully disclosed
Significant places	Residence/Workplace	0	Information about the location is not disclosed
		1	“Gu” of the building is disclosed
		2	“Dong” of the building is disclosed
			20–90 min on foot taken from a known location
		3	“Ro” or “Gil” of the building is disclosed
			4–20 min on foot taken from a known location
		3.5	<4 min on foot taken from a known location
		4	The exact location of the building is disclosed
Sensitive information	Hobby/Religion/Accomm.	0	Not disclosed
		1	Disclosed
Social relationship		0	No social relationship disclosed
		1	Only the relationship is disclosed
		2	The location and the relationship are disclosed together
Routine behavior		0	No place that is visited repeatedly
		1	Includes places that are visited repeatedly

#### 2.2.1. Demographics

The “Demographics” category included three sub-categories: Nationality, Gender, and Age. For Nationality and Gender, two scoring criteria were considered: (1) Level 0 for not containing any information for each of the two categories and (2) Level 1 for disclosing that information (e.g., Patient #5sx is Chinese, Patient #8nw is a woman). In the case of “Age,” three criteria were considered: (1) Level 0 for no age information, (2) Level 1 for rough description (e.g., the twenties), and (3) Level 2 for accurate information (e.g., 30 years old, born in 1990).

#### 2.2.2. Significant Places

Before describing the methods further, this study explains the administrative divisions in South Korea since it could differ from country to country. The administrative divisions can be divided into four levels by their size: province (“Do”; the whole country is composed of nine provinces), city (“Si”; typically 100–1,000 km2), sub-city (“Gu”; typically 10–,100 km2), and district (“Dong”; typically 1–10 km2) (19). People in South Korea often use this system when they look for a place or mention a certain location. In the address system of South Korea, there are two more detailed steps in describing places: streets (i.e., “Ro” or “Gil”) and the building number. The street is lower level than the “Dong,” so a “Dong” may contain several “Ro”s and “Gil”s. The lowest level is the building number and the address provided up to this step would point to the only building throughout the country.

A person's home (residence) and workplace are considered significant places. To assess the detailed location information of these places, a two-stage approach was used: (1) direct location identification and (2) indirect location inference by combining the breadcrumbs of visited places and transport modes.

**Figure 1:**
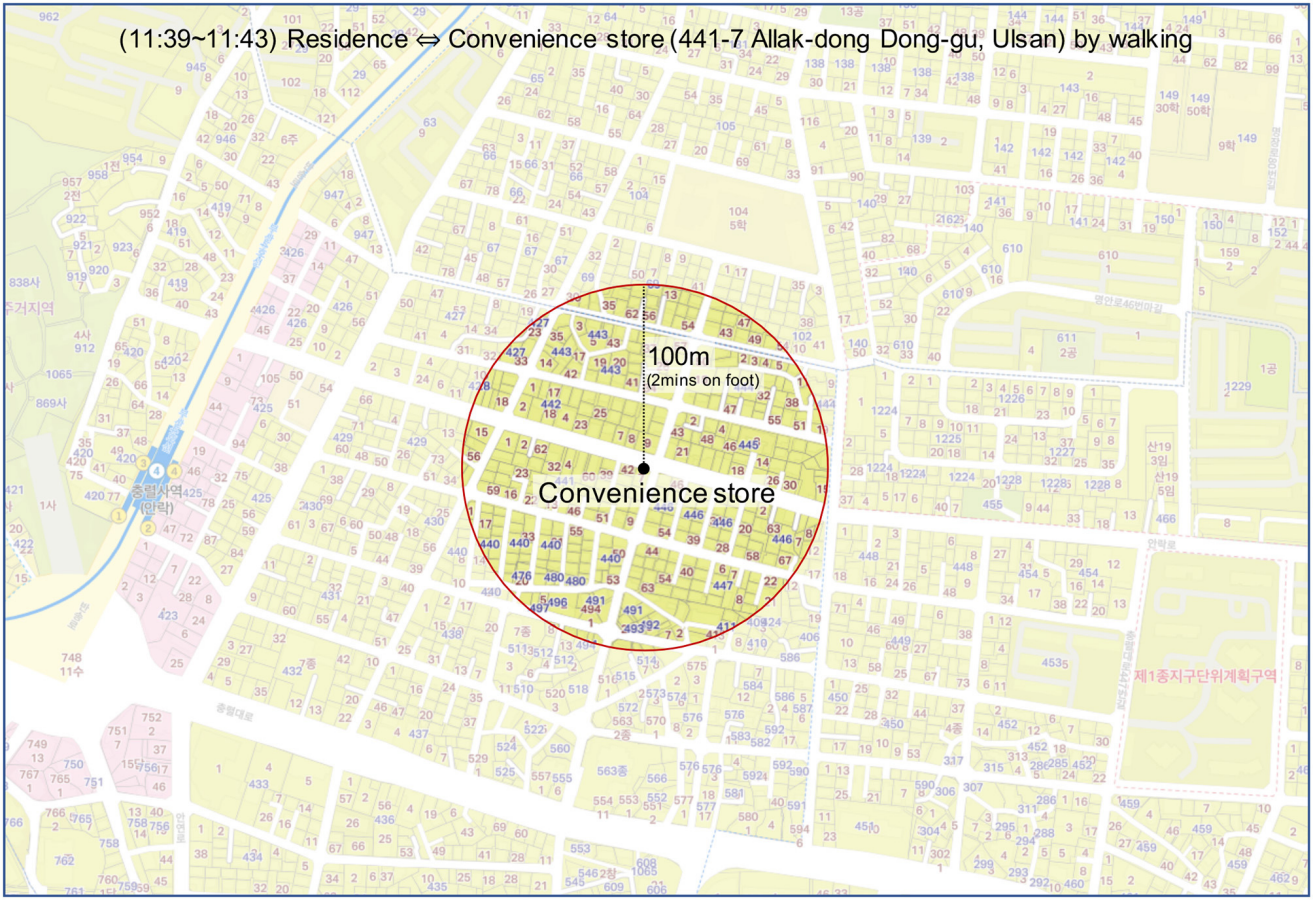
Inference of a patient's residence based on a travel log.

The second stage was inferring the locations of personal life using nearby places whose full addresses or names were disclosed. Even if the information is limited, reasonable inference based on a travel log is possible by examining the surrounding places and transport modes. For example, there was no explicit description of a patient's home, yet the travel log said “4 min in total to walk from his home to a convenience store, and come back again.” and the full address of the store is known (i.e., 441-7, Allak-dong, Dong-gu, Ulsan). This log may indicate the approximate location of her house. Considering a person's walking speed (e.g., 3 km/h), the area where her home is located could be determined as described in Figure 1.

To estimate the time required to travel on foot, the average sizes of the sub-city (“Gu”), district (“Dong”) and street (“Ro” or “Gil”) were used. There were 68 Gu, 1,033 Dong, and 41,301 Street included in the total for the seven cities. Given that the total size of these cities was 4,000 km2, the average sizes of Gu, Dong, and Street were calculated as 58.8, 3.9, and 0.1 km2, respectively. For the convenience of calculation, an assumption was made that the shape of each administrative area was circular. As a result, the radius of each division was 4.3, 1.1, and 0.2 km for Gu, Dong, and Street, respectively. Taking the average walking speed of a person as 3 km/h, the time required to travel the division on foot could be calculated. Consequently, it could be estimated that Gu, Dong, and Street take 90, 20, and 4 min to travel on foot, respectively. This means it is reasonable to infer that a place is under Dong level (i.e., privacy level 2) when it takes from 20 to 90 min on foot and Street level (i.e., privacy level 3) if it takes 4–20 min. On the basis of these results, the details of a location were labeled where the address was not shown but could be inferred from a known place. For instance, in the case of Patient #pr8 of BI1C-gu who went home from the Q5EG branch of KJN1 convenience store (i.e., only one store of its kind in that region) on foot in 5 min, this case was scored as level 3 privacy risk. Moreover, some places where it took <4 min on foot were labeled as 3.5. In this case, it is more specific than level 3, but it is still not possible to identify the exact place.

#### 2.2.3. Sensitive Places

In some buildings, there is a possibility of revealing sensitive personal information. For instance, if there is information on the travel log that the patient had attended a church service, and its name was disclosed, anyone who reads this could know her religion. This study mainly considered three place categories: (1) hobbies, such as fitness clubs, dance schools, PC cafes (playing games), and karaoke (singing); (2) religion, such as a church, cathedral, and temple; and (3) accommodation, such as hotel and motel. If any of these place categories were described in the travel log, that case was labeled as level 1; otherwise, level 0 was given.

#### 2.2.4. Social Relationship

Privacy issues might arise when information about how one person is related to another is revealed. If the travel log indicates that two people are found to have been together at a certain time or moved together to a place, there is privacy leakage of relationships. Therefore, patients' travel logs were examined to check whether they included this relationship information. For not describing such information, level 0 was given. Level 1 was rated in case of revealing the relationship only (e.g., Patient #t52 in 4XAL-gu is the mother-in-law of Patient #rb4 in the same district). If the relationship was revealed with location (e.g., Patient #90x in 8NUW-gu had lunch with her colleague Patient #v8l in the same district, at a restaurant near their office), that case was rated as level 2.

#### 2.2.5. Routine Behavior

Using information about places that are repeatedly visited in a specific time window (known as behavioral routines) could make it easier to identify a person. If it is revealed that there is a place where a confirmed patient repeatedly visits at a certain time, malicious people may use this information (e.g., robbery). For this reason, it was examined whether the travel log included routine behavior. If there was a place visited more than twice at a specific time, the case was labeled as a level 1 risk, otherwise, a level 0 risk (or no risk at all).

## 3. Results

This study analyzes 970 cases from seven metropolitan cities in South Korea (see Table 4) and reports (1) the descriptive statistics of privacy risk levels, and (2) their differences across regions and time.

**Table 4:** Number of confirmed patients across regions.

**Total**	**Region**						
	**Seoul**	**Busan**	**Incheon**	**Sejong**	**Ulsan**	**Daejeon**	**Gwangju**
970	591	129	92	46	43	39	30

### 3.1. Patterns of Privacy Risk Levels

The five major categories and eight sub-categories of data types that might potentially reveal personal information (e.g., life cycle, social relationships, etc.) were coded in terms of privacy risk levels. Here, a detailed description of the result as well as some noteworthy findings from the analysis of the privacy risk of the contact trace data is provided (see Table 5).

**Table 5:** The average of privacy levels across regions. Values in brackets indicate standard deviation.

**Category**	**Sub-cat**.	**Privacy level**							
		**Overall**	**Regions**						
			**Seoul**	**Busan**	**Incheon**	**Sejong**	**Ulsan**	**Daejeon**	**Gwangju**
Demographics	Nationality	0.09 (0.28)	0.07 (0.25)	0.04 (0.19)	0.10 (0.30)	0.00 (0.00)	0.02 (0.15)	0.00 (0.00)	1.00 (0.00)
	Gender	1.00 (0.00)	1.00 (0.00)	1.00 (0.00)	1.00 (0.00)	1.00 (0.00)	1.00 (0.00)	1.00 (0.00)	1.00 (0.00)
	Age	1.86 (0.34)	1.97 (0.18)	2.00 (0.00)	2.00 (0.00)	1.02 (0.15)	2.00 (0.00)	1.00 (0.00)	1.07 (0.25)
Sig. places	Residence	1.96 (0.69)	2.11 (0.55)	1.30 (0.66)	1.83 (0.55)	3.00 (0.21)	1.21 (0.47)	2.26 (0.64)	1.37 (0.72)
	Workplace	0.93 (1.61)	0.78 (1.49)	0.72 (1.52)	1.32 (1.77)	3.11 (1.64)	0.44 (1.18)	1.41 (1.92)	0.40 (1.10)
Sensitive info.	Hobby	0.07 (0.26)	0.07 (0.25)	0.06 (0.24)	0.05 (0.23)	0.17 (0.38)	0.09 (0.29)	0.08 (0.27)	0.07 (0.25)
	Religion	0.11 (0.32)	0.06 (0.24)	0.36 (0.48)	0.04 (0.21)	0.02 (0.15)	0.30 (0.46)	0.05 (0.22)	0.27 (0.45)
	Accomm.	0.02 (0.12)	0.02 (0.12)	0.02 (0.15)	0.03 (0.18)	0.00 (0.00)	0.00 (0.00)	0.00 (0.00)	0.00 (0.00)
Social relationships		0.75 (0.85)	0.85 (0.86)	0.44 (0.74)	0.60 (0.79)	0.46 (0.78)	1.23 (0.84)	0.28 (0.56)	0.97 (0.85)
Routine behavior		0.24 (0.43)	0.22 (0.41)	0.24 (0.43)	0.25 (0.44)	0.46 (0.50)	0.21 (0.41)	0.38 (0.49)	0.17 (0.38)

*Sub-cat., sub-category; sig., significant; info., information; accomm., accommodation*.

#### 3.1.1. Demographics

Demographics included patients' nationality, gender, and age. In reporting nationality, 91.2% of the data do not contain patients' nationality (*n* = 885). These cases could be assumed to be Koreans. All cases of confirmed foreign expatriates disclosed their nationality, which accounted for 8.8% (*n* = 85) of the patients. Considering that legal foreign expatriates account for only 4% of South Korea's total population (20), and the number of confirmed foreign cases is a small proportion, there is a high chance of identifying an individual: it is easier to pinpoint an individual if cases from his/her nationality are relatively few. For example, there was only one confirmed case from Gambia, while ~260 Gambians resided in South Korea. This example shows the potential for easier identification of the suspect when the size of a community is small.

All cases reported patients' gender, and 839 cases (86.5%) specified the exact age or birth year of a patient (e.g., age 30, born in 1990), whereas 131 cases (13.5%) only reported the age range of a patient (e.g., the twenties). One thing to note is that age and gender are personal details that make up one's social security numbers (3 digits) and collecting such data could be invasion of privacy.

#### 3.1.2. Significant Places

Significant places refer to the residence and workplace of an individual. In identifying residence, over 70% (*n* = 759) of the disclosed data ranging from level 2 to level 4 provide highly granular data, such as the district, street, and name of an apartment. With additional data, such as activity type (e.g., walking) and the time taken, it could easily be deduced that an individual lives in that narrowly defined region. Only 15 cases were labeled as level 0, which included the following two cases: (1) patients from abroad with no domestic residence, and (2) patients who had come from another city. Of the disclosed data, 22.3% (*n* = 216) ranged from level 3 to 4 in the “Workplace” category. One interesting fact to note was that collective infection at a workplace unavoidably revealed a patient's workplace location. For example, a collective infection case which caused about 118 related cases occurred at a call center located in Guro-gu, Seoul revealed the specific building and floor of the center (e.g., “Korea” building, 11th floor). A large fraction of cases had a level 0 on workplace location (*n* = 703, 72.5%). This low risk of workplace location is possibly due to the confirmed patients being jobless (e.g., older adults, teenagers, patients from abroad). Another noteworthy finding is that collective infection at a workplace inevitably exposes the location and the patient's job, which the patient wished to keep private (e.g., Patient #u9m from 73TB-gu, Seoul, works in the redlight district). Other cases classified as “No information” usually had no related information of a workplace. Some exceptional cases included the word “office,” but with no location specified (e.g., 9 a.m.–6 p.m., office).

#### 3.1.3. Sensitive Information

The data revealed several cases of patients' regular visits to a certain place, which makes it possible to infer one's personal details—hobby, religion, and accommodation information. In the hobby category, 69 cases (*n* = 69, 7.1%) were identified from patients' regular visits to the gym, golf club, and other places for amusement or leisure activities (see Table 6). Furthermore, religious orientations were revealed because of the collective infection that occurred through religious activities, such as group prayers (*n* = 111, 11.4%). After mass contagion, most religious services went online, and only a few infection cases revealed religious places. It was also found that information of a short stay (e.g., a few hours) at a specific accommodation, hotel, or motel, may infringe privacy—although this constituted only a small proportion (*n* = 15, 1.6%).

**Table 6:** The percentage of privacy levels across regions.

**Category**	**Sub-cat**.	**Privacy levels**	**Overall**	**Region**						
				**Seoul**	**Busan**	**Incheon**	**Sejong**	**Ulsan**	**Daejeon**	**Gwangju**
Demographics	Nationality	0	91.2	93.2	96.1	90.2	100.0	97.7	100.0	0.0
		1	8.8	6.8	3.9	9.8	0.0	2.3	0.0	100.0
	Gender	0	0.0	0.0	0.0	0.0	0.0	0.0	0.0	0.0
		1	100.0	100.0	100.0	100.0	100.0	100.0	100.0	100.0
	Age	0	0.0	0.0	0.0	0.0	0.0	0.0	0.0	0.0
		1	13.5	3.2	0.0	0.0	97.8	0.0	100.0	93.3
		2	86.5	96.8	100.0	100.0	2.2	100.0	0.0	6.7
Sig. places	Residence	0	3.0	1.7	0.0	2.2	0.0	0.0	0.0	10.0
		1	18.8	3.7	80.6	18.5	0.0	81.4	10.3	46.7
		2	59.9	78.0	8.5	73.9	2.2	16.3	53.8	40.0
		3	16.0	12.9	10.9	5.4	95.7	2.3	35.9	3.3
		3.5	2.3	3.7	0.0	0.0	0.0	0.0	0.0	0.0
		4	0.1	0.0	0.0	0.0	2.2	0.0	0.0	0.0
	Workplace	0	72.5	75.5	81.4	60.9	21.7	86.0	64.1	86.7
		1	3.1	4.6	0.0	2.2	0.0	2.3	0.0	0.0
		2	2.2	1.9	0.0	8.7	0.0	0.0	0.0	6.7
		3	5.5	2.5	2.3	1.1	2.2	4.7	2.6	0.0
		3.5	0.3	0.5	0.0	0.0	0.0	0.0	0.0	0.0
		4	16.5	15.1	16.3	27.2	76.1	7.0	33.3	6.7
Sensitive info.	Hobby	0	92.9	93.4	94.1	94.6	82.6	91.3	92.3	93.7
		1	7.1	6.6	5.9	5.4	17.4	8.7	7.7	6.3
	Religion	0	88.6	93.8	65.9	95.7	97.8	71.7	94.9	75.0
		1	11.4	6.2	34.1	4.3	2.2	28.3	5.1	25.0
	Accomm.	0	98.4	98.5	97.8	96.7	100.0	100.0	100.0	100.0
		1	1.6	1.5	2.2	3.3	0.0	0.0	0.0	0.0
Social relationship		0	51.3	45.3	70.5	58.7	71.7	25.6	76.9	36.7
		1	22.2	24.2	14.7	22.8	10.9	25.6	17.9	30.0
		2	26.5	30.5	14.7	18.5	17.4	48.8	5.1	33.3
Routine behavior		0	75.9	78.0	76.0	75.0	54.3	79.1	61.5	83.3
		1	24.1	22.0	24.0	25.0	45.7	20.9	38.5	16.7

*Sub-cat., sub-category; sig., significant; info., information; accomm., accommodation*.

#### 3.1.4. Social Relationship

Along with location data, some of the patients' relationship information was also provided. With relationship data alone or combining location and relationship data, it might be possible to guess a patient's social boundaries and even infer more about personal life. Thus, the category was divided into “Relationship only” and “Relationship and Location.”

In “Relationship only” (*n* = 215, 22.2%), family and social relations (e.g., colleagues, friends) of a patient were identified. From the analysis, the disclosure of family relations was shown to contain the following two categories: (1) disclosure of family information involving consecutive infection of family members (e.g., Patient #8dj (Seoul) mother from Daegu visited Patient #8dj's house, Patient #t5v (Seoul) Patient #8dj's sister), and (2) disclosure of information on an uninfected family member (e.g., Patient #sa3 (Seoul) Patient #sa3's husband had contact with Patient #x6t at work and she was infected while under self-quarantine). In the first category, it was found that information about family relations was usually provided directly as family members' traces overlap and involve consecutive infections. The second category raises questions on the necessity of providing additional information about an uninfected family member. For example, information from the second case unnecessarily reveals that the patient's husband had contact with another patient who was assumed to be his colleague. Considering that the patient's husband was not infected, it is difficult to say if his contact with a colleague was an essential piece of information.

Compared to family relations, social relations of confirmed cases generally provide activities shared together (e.g., carpool, late-night drinks at the bar). In the case of workplace relations, linkage information between patients was revealed largely through collective infection. Some cases revealed additional information other than a colleague/friend relationship. For example, contact trace data of Patient #9f5 (Seoul) revealed his colleague is a member of D0L6 church, a church that was identified as the epicenter of the major outbreak in South Korea after the infection of Patient #f61, a “super-spreader” from Daegu. The local government may have judged that providing this information was necessary considering the severity of the outbreak situation. However, the question still remains as to whether it was an appropriate decision to disclose information about religion along with social relationships.

“Relationship and Location” (*n* = 257, 26.5%) provides information on visits to certain places that may reveal the presence of another person and lead to speculation and unwanted exposure of one's private relationship. For example, one patient's repeated visits to a motel at regular intervals may lead to speculation that he has an intimate relationship with someone. Although excluded from our data analysis, Patient #f24 from Suwon (one of the cities in South Korea) who had his traces overlapped with his sister-in-law (Patient #8if) was highly criticized by the media and social network for having an affair, which turned out to be a rumor (21). Less sensitive cases reported the location of home and workplace of a patient's family, friends, and other acquaintances.

#### 3.1.5. Routine Behavior

From the data, it was able to identify types of frequent activities of a patient (e.g., commuting, exercise), which extends to inference on a patient's routine behavior and lifestyle patterns (*n* = 234, 24.1%). For example, ~55% of the contact trace data from Seoul reported regular commuting time of the patients. These pieces of information are usually provided along with the type of transportation (e.g., on foot/by car/by bus/carpool with a colleague), which enables a detailed inference on one's time schedule. Data of patient #t2n (Seoul) showed repetitive commuting to a church and his later mobility patterns centered around the church. The patient also visited a nearby cafe several times at a similar time before the case was confirmed. This consistent pattern leads us to a plausible speculation that he is a Christian who works at a church and often visits nearby places. The speculation in this study was confirmed through a news article that revealed his job, a missionary. As the high data granularity provided in this case leads to several assumptions on private information, it was found that inferred details of the patient (workplace, frequent visits, religion) could also belong to other categories, such as “Significant Places” and “Sensitive Information.”

Key findings

- Demographics were observed in most cases (gender: 100%, age: 86.5%) and the data on significant places (residence/workplace) showed different levels of privacy risks in over 70% of the cases.
- Some places disclosed in the data indicated sensitive information about the patient due to the characteristics of the place (e.g., PC caf'e —the patient's hobby is playing games, church—the patient is Christian). In addition, nearly half of the cases (48.7%) exposed the patient's social relationships by describing information about relationships or by showing them visiting certain places with others.
- Around a quarter of the cases (24.1%) revealed the routine behavior of the patient from places that had been visited repeatedly and frequently. The patterns that appeared in routine behavior may be an important factor in inferring the patient's lifestyle.

### 3.2. Patterns of Data Disclosure Levels Across Regions and Over Time

#### 3.2.1. Difference in Data Disclosure Across Regions

First, variation in privacy risk levels across different regions was analyzed by comparing their average privacy levels. The analyses revealed regional differences in privacy risks for the confirmed patients.

In the demographics category, four cities, Seoul, Busan, Incheon, and Ulsan, often showed the exact age of patients (e.g., 27 years; i.e., level 2), while Sejong, Daejeon and Gwangju showed the age range (e.g., the twenties; i.e., level 1). In terms of nationality, Seoul disclosed the nationalities of the confirmed cases of all foreigners. Despite its low proportion (~7%) relative to the number of total cases, Seoul reported a higher number of nationalities compared to other cities. It was posited that this was because of capital-specific effects, as the city has ~400,000 foreigners. Gwangju also reported a considerably high number of nationalities. Out of the total 30 cases, Gwangju revealed nationality information of all the cases (100% disclosure). Unlike Seoul, one interesting fact to note from Gwangju is that the city also reported the nationality of Korean patients. Currently, no specific guidelines regarding nationality disclosure have been found. As shown earlier, all cities revealed gender information of the patients, and there was no difference in this regard.

In addition, a comparison of the privacy level of significant places was conducted. As shown in Table 5, the average privacy level of residence is distributed between 1.21 (Ulsan) and 3.00 (Sejong). All the cities except Sejong released only approximate information on a patient's residence such that more than half of the residential information released by each city was equal to or below level 2 (“Dong” level). Sejong revealed the most detailed information with level 3 on average (mostly at an apartment complex level), which is partly because of the unique characteristics of Sejong, a new multifunctional administrative city with many high-rise apartment buildings.

With regard to the workplace, the presence of a mass infection in the same building made the difference. Important cases, such as the call center of an insurance company in Guro-gu, Seoul, influenced the high proportion of level 4 cases in Seoul (15.1%) and Incheon (27.2%); same was the case with a government building of the Ministry of Oceans and Fisheries in Sejong (76.1%). Most of the patients in Sejong work in government buildings, thereby resulting in a high ratio of level 4. Daejeon showed a comparatively high ratio of level 4 (33.3%), despite having no case of mass infection, unlike other cities.

In the “Sensitive Information” category, “Hobby” showed a substantial proportion of cases that reported privacy level 0 across all cities. In level 1, Sejong reported 17.4%, which is a markedly higher figure compared to other cities. This is interesting to note, as one patient who took a Zumba class infected the other students. “Religion” showed a moderately high percentage of level 0 in an overall sense, but Busan showed 34.1% of cases that were level 1. Collective infection occurred at a church that contributed to this relatively high level of disclosure. “Accommodation” information appeared only in a small fraction of the dataset, but such visits were often suspected for cheating, as reported in the news articles (10). From “Hobby” and “Religion,” it was found that a particular incident that involved collective infection unavoidably led to a disclosure of sensitive information.

“Routine Behavior” showed a higher average level of disclosure than “Sensitive Information.” In this category, Sejong and Daejeon showed relatively high percentages of 45.7 and 38.5%, respectively. In Sejong (*n* = 46), confirmed cases showed very similar mobility patterns, as collective infection revealed that most of the patients worked at the same government and shared the same leisure activity (i.e., Zumba class). It was assumed that the unique characteristics of this newly built administrative city have also contributed to this dense infection within the community, as the population is relatively small and a large proportion of residents are government officials. Despite no occurrence of collective infection, Daejeon (*n* = 39), as shown earlier, revealed the workplace of the confirmed patients. Disclosed workplaces are usually research institutes or tech companies, as the city is a well-known mecca of science and technology in South Korea. From the data, 84.6% of workplace revelations were particularly found in Seo-Gu and Yuseong-Gu, districts dense with research institutes. Inferring the patients' routine behavior was relatively easier as their workplaces were revealed and they lived in the same area. Cases from these two cities demonstrate that characteristics of a city can be reflected in contact trace data and enable an indication of one's routine behavior and daily patterns.

In “Social Relationship,” Ulsan showed the highest percentage of data disclosure (level 1 and level 2 combined: 72.4%), followed by Gwangju (level 1 and level 2 combined: 63.3%). From Ulsan, it was posited that mass influx from abroad and their traces with family members may have contributed to this high percentage of privacy disclosure.

#### 3.2.2. Difference in Data Disclosure by the Provision of Guidelines

The Korean government announced a guideline limiting the scope and detail of the information to be disclosed on March 14, 2020. As shown in Table 7, it was analyzed how the release of the government's official guidelines influenced privacy risk levels across different regions, by comparing the average privacy levels before and after the announcement.

**Table 7:** The average of privacy levels before and after a guideline for contact tracing data (March 14).

**Category**	**Sub-cat**.	**Guideline (Mar. 14)**	**Overall**	**Regions**						
				**Seoul**	**Busan**	**Incheon**	**Sejong**	**Ulsan**	**Daejeon**	**Gwangju**
Demographics	Nationality	Before	0.07 (0.25)	0.06 (0.24)	0.01 (0.10)	0.07 (0.26)	0.00 (0.00)	0.00 (0.00)	0.00 (0.00)	1.00 (0.00)
		After	0.10 (0.31)	0.07 (0.26)	0.13 (0.34)	0.11 (0.31)	0.00 (0.00)	0.06 (0.25)	0.00 (0.00)	1.00 (0.00)
	Gender	Before	1.00 (0.00)	1.00 (0.00)	1.00 (0.00)	1.00 (0.00)	1.00 (0.00)	1.00 (0.00)	1.00 (0.00)	1.00 (0.00)
		After	1.00 (0.00)	1.00 (0.00)	1.00 (0.00)	1.00 (0.00)	1.00 (0.00)	1.00 (0.00)	1.00 (0.00)	1.00 (0.00)
	Age	Before	1.84 (0.37)	2.00 (0.00)	2.00 (0.00)	2.00 (0.00)	1.03 (0.16)	2.00 (0.00)	1.00 (0.00)	1.13 (0.35)
		After	1.89 (0.32)	1.95 (0.22)	2.00 (0.00)	2.00 (0.00)	1.00 (0.00)	2.00 (0.00)	1.00 (0.00)	1.00 (0.00)
Sig. places	Residence	Before	1.95 (0.78)	2.10 (0.63)	1.37 (0.71)	1.89 (0.57)	2.97 (0.16)	1.26 (0.53)	2.59 (0.59)	1.27 (0.59)
		After	1.98 (0.60)	2.12 (0.50)	1.10 (0.40)	1.80 (0.54)	3.13 (0.35)	1.13 (0.34)	1.82 (0.39)	1.47 (0.83)
	Workplace	Before	1.50 (1.84)	1.46 (1.79)	0.79 (1.57)	3.00 (1.61)	3.24 (1.57)	0.67 (1.44)	1.77 (2.00)	0.80 (1.47)
		After	0.44 (1.18)	0.37 (1.09)	0.52 (1.36)	0.58 (1.26)	1.63 (1.77)	0.06 (0.25)	0.94 (1.75)	0.00 (0.00)
Sensitive info.	Hobby	Before	0.12 (0.33)	0.13 (0.33)	0.08 (0.28)	0.11 (0.31)	0.21 (0.41)	0.15 (0.36)	0.14 (0.35)	0.13 (0.35)
		After	0.03 (0.16)	0.03 (0.17)	0.00 (0.00)	0.03 (0.18)	0.00 (0.00)	0.00 (0.00)	0.00 (0.00)	0.00 (0.00)
	Religion	Before	0.19 (0.39)	0.07 (0.25)	0.45 (0.50)	0.07 (0.26)	0.03 (0.16)	0.48 (0.51)	0.09 (0.29)	0.47 (0.52)
		After	0.05 (0.22)	0.06 (0.24)	0.06 (0.25)	0.03 (0.18)	0.00 (0.00)	0.00 (0.00)	0.00 (0.00)	0.07 (0.26)
	Accomm.	Before	0.01 (0.11)	0.01 (0.12)	0.03 (0.17)	0.00 (0.00)	0.00 (0.00)	0.00 (0.00)	0.00 (0.00)	0.00 (0.00)
		After	0.02 (0.13)	0.02 (0.13)	0.00 (0.00)	0.05 (0.21)	0.00 (0.00)	0.00 (0.00)	0.00 (0.00)	0.00 (0.00)
Social relationship		Before	0.79 (0.86)	1.03 (0.87)	0.36 (0.66)	0.54 (0.84)	0.39 (0.72)	1.37 (0.84)	0.36 (0.58)	1.00 (0.76)
		After	0.72 (0.83)	0.74 (0.83)	0.71 (0.90)	0.63 (0.77)	0.75 (1.04)	1.00 (0.82)	0.18 (0.53)	0.93 (0.96)
Routine behavior		Before	0.36 (0.48)	0.35 (0.48)	0.29 (0.45)	0.46 (0.51)	0.53 (0.51)	0.26 (0.45)	0.41 (0.50)	0.33 (0.49)
		After	0.14 (0.35)	0.14 (0.35)	0.10 (0.30)	0.16 (0.37)	0.13 (0.35)	0.13 (0.34)	0.35 (0.49)	0.00 (0.00)

*Values in brackets indicate standard deviation. Sub-cat., sub-category; sig., significant; info., information; accomm., accommodation*.

Overall, average privacy risk levels decreased for the workplace, hobby, religion, and routine behavior, whereas other items remained somewhat similar. It is notable that while detailed demographic information (i.e., nationality, gender, and age) is generally considered as sensitive information, the average privacy levels for these remained unchanged even after the announcement.

In privacy risk levels in general, every region showed a similar the change in trend. However, notable regional differences were found in accommodation and relationships; as an illustration, for relationships, the average levels decreased for Seoul, Daejeon, and Gwangju, while the levels increased for Busan and Sejong.

These findings indicate that the announcement of government guidelines can lower risk levels. However, the effects of the government guidelines could be limited to several factors, such as workplaces and routine behaviors, and vary across regions (or local governments).

Key findings

- Differences in privacy risk levels among the cities were observed. In particular, the data from Sejong revealed the most detailed information on significant places (the average privacy risk levels for residence and workplace in Sejong were over level 3), whereas Ulsan showed a relatively high percentage of data disclosure on social relationships (i.e., 72.4% of the confirmed patients in Ulsan).
- The government guidelines on data disclosure have been released recently, and the effects were limited to a few factors, such as workplaces and routine behaviors.

## 4. Discussion

### 4.1. Not Too Much, Not Too Little: Seeking Just the Right Amount of Information Disclosure

Disclosed contact trace data (e.g., “where, when, and for how long”) help people to self-identify potential close contacts with people confirmed to be infected. However, *location trace disclosure* may pose privacy risks because a person's significant places and routine behaviors can be inferred. Privacy risks are largely dependent on a person's mobility patterns, which are affected by several regional and policy factors (e.g., residence type, nearby amenities, and social distancing orders). In addition, the results showed that disclosed contact trace data in South Korea often include *superfluous information*, such as detailed demographic information (e.g., age, gender, nationality), social relationships (e.g., parents' house), and workplace information (e.g., company name). Disclosing such personal data of already *identified* persons may not be useful for contact tracing whose goal is to locate *unidentified* persons who may be in close contact with confirmed people. In other words, for contact tracing purposes, it would be less useful to disclose the personal profile of the confirmed person and their social relationships, such as family or acquaintances. The detailed location of the workplace could be omitted because, in most cases, it is easy to reach employees through internal communication networks; an exceptional case would be when there is a concern of potential group infection with secondary contagions. Likewise, it is not necessary to reveal detailed travel information of overseas entrants (which were not reported in the main results), such as the arrival flight number and purpose/duration of foreign travels.

### 4.2. Policy and Technical Implications

Based on the results and discussions, this subsection presents policy and technical implications for contact tracing and data disclosure.

#### 4.2.1. Policy Implications

*Detailed guidelines are required*: The scope and details of patient data disclosure should be carefully considered in the official guidelines. As shown earlier, some of the information included in the patient data in South Korea could be controversial because it is not clear whether it is essential to prevent further spread of COVID-19. The current guidelines set by the KCDC, which are shown in Table 1, do not provide detailed recommendations. Therefore, the guideline about “information that identifies a specific person” could be interpreted differently by different contact trace officers. At the time of contact tracing, it is difficult for officials to envision how a combination of different pieces of information provides an important clue the patient's identity. To reduce the possibility of subjective interpretation, current guidelines can be augmented with the patterns of problematic disclosure, which could be documented by carefully reviewing existing cases. In this case, the codebook of this study could serve as a starting point for analyzing the patterns of problematic disclosure. For instance, one's residence and workplaces can be generally considered sensitive information. The codebook allows the assessment of privacy risk level on a patient's residence and workplaces when disclosing the patients' visited places and transport modes. In addition, for location privacy protection, privacy protection rules, such as *k*-anonymity can be applied. The *k*-anonymity ensures that *k* people in that region cannot be distinguished (22). Due to public safety, however, its strict application is not feasible, yet a relaxed version of *k*-anonymity can be used: at least for a given region, when there are multiple confirmed cases with overlapping periods, removing identifiers (or confirmed case numbers) could be considered to further protect their location privacy.

*Proper management of revealed data is required:* Given that some level of privacy risk is unavoidable due to public safety, it is important to manage the patients' data that have been opened to the public. Official guidelines recommend that municipalities erase outdated data from their official websites. While scouring the dataset over several months for this research, it was noticed that contact trace data are replicated on multiple sources, ranging from official channels of municipalities (e.g., homepage, blogs, social media, and debriefing videos on YouTube) to online news articles and personal sites. Diversifying information access channels would be beneficial for public safety; however, the authorities should set a strict code of conduct or regulations on *managing* replicated contact trace data (e.g., “register before publish”) to promote responsible use (e.g., removing outdated data).

#### 4.2.2. Technical Implications

*It's possible to automatically check privacy issues*: Contact tracers' subjective interpretation could be a source of privacy risks. One could consider an intelligent system that detects possible privacy issues from the patient data before disclosure. For example, personal data can be detected by utilizing supervised machine learning that analyzes semantic, structural, and lexical properties of the data (23) or by estimating privacy risks with visual analytic tools based on *k*-anonymity and *l*-diversity models (22). If a system utilizes a metric for quantifying the privacy threat and evaluation model as proposed in the previous study (24), the system could not only detect potential issues but also obscure the data automatically until it meets a certain privacy level. However, these automatic approaches should be considered with care because they may hide essential contact trace information that needs to be released for public safety.

*Unified management of contact tracing data could be introduced*: Decentralized management of contact trace data in each municipality makes it difficult to examine privacy risks and manage data replication. In addition, the quality of user interfaces varies widely across different regions. Introducing a unified system that manages and visualizes the contact trace data across all regions would be beneficial. Of course, there is a concern of a single point of failure, yet this issue can be overcome by introducing decentralized server systems with cloud computing. To promote responsible replication and management of patient data, one can implement a “register before publish” policy. Moreover, an information system can help to manage the people who reprocess the patient data officially provided by the local government and deliver it to the public via news articles. This system should have the ability to (1) authorize data usage, (2) track in which article the data is being used, and (3) delete the data automatically when it is outdated. The system could also provide a built-in sharing feature as in YouTube's video embedding. YouTube allows users to add a video to their websites, social network sites, and blogs by embedding the video to the sites, while any modification or deletion of the original video on YouTube is also reflected in the embedded video (25). A similar mechanism can also be applied to the system.

*Mobile technologies for contact tracing can be alternatively considered*: Mobile technologies could be utilized to avoid privacy concerns from public disclosure (26, 27). Short-range wireless communications (Bluetooth) can be used to automatically detect close contacts by keeping periodic scanning results of nearby wireless devices [e.g., TraceTogether (28) and Apple/Google's app (29, 30)]. A confirmed user can now publish its anonymized Bluetooth ID, which helps other people to check whether they are in close contact with the patient. This approach certainly helps protect user privacy because location information is not explicitly shared. However, there are major concerns about its assumption: a majority of people voluntarily need to install mobile applications. There should be further studies on how to consider multiple contact tracing methods along with traditional methods of public disclosure.

### 4.3. Limitations and Future Work

With the outbreak of COVID-19, as mentioned in the introduction, several countries have been disclosing contact trace data. Although this paper presents the privacy risks of contact tracing practices, the results should be carefully interpreted, given the limitations of the study. First, this work is focused on South Korea and the results may not be generalizable to other nations due to policy differences. However, our methodologies and insights could still be applied in other nations that make contact trace data public. Comparing the differences in disclosure policies and privacy risk levels would be an interesting direction for future work, as slight differences in disclosure exist. For instance, the Hong Kong government reveals the patient's information in an interactive map dashboard that showed not only the demographics but also the full address of both residential and non-residential places that the patient had visited (31). The Singapore government also released detailed patient information, such as nationality, visited sites, and infection sources (4). Aggressive contact tracing and data disclosure were considered effective methods for suppressing the spread of a virus. While there is an ongoing dispute between promoting public safety and protecting personal privacy, there is a growing consensus that a reasonable level of personal privacy needs to be sacrificed for public safety, as shown in a recent survey (32). For all these cases, our policy and technical implications could help lower privacy risks and yet allow governments to effectively conduct contract tracing. In future studies, researchers could compare the differences between governmental policies of open access to contact trace data and the opinions from the public among these countries to set international guidelines on data disclosure in pandemic situations.

Next, there are privacy issues that remain to be quantified; for example, revealing foreign travel logs, underlying medical conditions, and even part of a patient's name (i.e., the last name of the patient). Place log information may include hospital visits that are not related to COVID-19; this could reveal a patient's underlying health or personal conditions (e.g., urology, dermatology, and cosmetic surgery). Therefore, this study should be expanded to evaluate diverse privacy-violating elements. It is also necessary to study the media's disclosure patterns of patient information. In some cases, the media provided more specific data than the government through an exclusive report. Recently in South Korea, new media publicized a patient's sexual orientation by investigating visited places (e.g., specific types of bars) or workplace/social information (e.g., infected healthcare workers). Therefore, one could compare the disclosed data from the local government with that from the media to evaluate how much further privacy leakage would occur through the news media. This work mainly focused on analyzing the officially disclosed patient data, nevertheless, it is also important to find out what people (both patients and the public) really think about that data. Opinions on sharing *my data* as opposed to *someone else*'s may differ (33), and the perception of risk of information disclosure could be influenced by the consequent results of both benefits and risks (34). Thus, researchers could possibly find an optimal level where personal privacy and public benefit are well-balanced.

## Data Availability Statement

All datasets presented in this study are included in the article/ supplementary material.

## Author Contributions

GJ and HL collaboratively analyzed the dataset and wrote the main texts (i.e., Introduction, Results, Discussion). AK actively guided the design of the study, helped data analyses/visualizations, and wrote the background and summary sections. UL supervised the overall research, provided detailed feedback for data analyses and paper organization, and reviewed the entire manuscript. All authors contributed to the article and approved the submitted version.

## Conflict of Interest

The authors declare that the research was conducted in the absence of any commercial or financial relationships that could be construed as a potential conflict of interest.
